# Non-Invasive Detection and Staging of Colorectal Cancer Using a Portable Electronic Nose

**DOI:** 10.3390/s21165440

**Published:** 2021-08-12

**Authors:** Heena Tyagi, Emma Daulton, Ayman S. Bannaga, Ramesh P. Arasaradnam, James A. Covington

**Affiliations:** 1 School of Engineering, University of Warwick, Coventry CV4 7AL, UK; Heena.tyagi@warwick.ac.uk (H.T.); e.daulton@warwick.ac.uk (E.D.); 2 Department of Gastroenterology, University Hospital Coventry & Warwickshire, Coventry CV2 2DX, UK; ayman.bannaga@warwick.ac.uk (A.S.B.); r.arasaradnam@warwick.ac.uk (R.P.A.); 3 Warwick Medical School, University of Warwick, Coventry CV4 7AL, UK; 4 School of Health Sciences, Coventry University, Coventry CV1 5FB, UK; 5 Leicester Cancer Centre, University of Leicester, Leicester LE1 7RH, UK

**Keywords:** electronic nose, colorectal cancer, PEN3, GC-TOF-MS, VOCs

## Abstract

Electronic noses (e-nose) offer potential for the detection of cancer in its early stages. The ability to analyse samples in real time, at a low cost, applying easy–to-use and portable equipment, gives e-noses advantages over other technologies, such as Gas Chromatography-Mass Spectrometry (GC-MS). For diseases such as cancer with a high mortality, a technology that can provide fast results for use in routine clinical applications is important. Colorectal cancer (CRC) is among the highest occurring cancers and has high mortality rates, if diagnosed late. In our study, we investigated the use of portable electronic nose (PEN3), with further analysis using GC-TOF-MS, for the analysis of gases and volatile organic compounds (VOCs) to profile the urinary metabolome of colorectal cancer. We also compared the different cancer stages with non-cancers using the PEN3 and GC-TOF-MS. Results obtained from PEN3, and GC-TOF-MS demonstrated high accuracy for the separation of CRC and non-cancer. PEN3 separated CRC from non-cancerous group with 0.81 AUC (Area Under the Curve). We used data from GC-TOF-MS to obtain a VOC profile for CRC, which identified 23 potential biomarker VOCs for CRC. Thus, the PEN3 and GC-TOF-MS were found to successfully separate the cancer group from the non-cancer group.

## 1. Introduction

Cancer remains a leading cause of death worldwide, with approximately 19.3 million new cases and 10 million deaths in 2020 [1]. Survival rates depend on early detection; however, many current methods do not provide the means to achieve this or are not applied [2]. One potential method to support cancer detection is through the measurement of Volatile Organic Compounds (VOCs) that reflect the biological process of disease. These bodily VOCs are the reflection of the physiological effects and metabolism of the individual and the environment surrounding them. They are generated as the products of the biological activities inside the body and can be released from saliva, urine, breath, blood, or faeces [3,4,5,6]. Cancer causes changes in these biological pathways leading to the emission or omission of specific VOCs [7].

Colorectal cancer (CRC) is the second leading cause of the cancer-related deaths and third leading cause of cancer-related deaths among men and women, respectively, in Europe [8]. As such, screening using faecal immunochemical testing for haemoglobin (FIT) has been introduced but still can miss up to 10% of cancers [9,10,11]. More recently, there is evidence to suggest addition of VOC to FIT can further improve diagnosis of CRC [12]. Colonoscopy is considered the most reliable way of detecting CRC at both early and advanced stages, but the rate of detection depends upon the operator performing the procedure. Furthermore, colonoscopy is an invasive, expensive, and uncomfortable procedure with a small risk of bowel injury [13,14].

Several techniques are available for the detection and analysis of VOCs. Gas Chromatography–Mass Spectrometry (GC-MS) is considered as the gold standard method for the detection of VOCs with high sensitivity and specificity. However, these analytical methods may be unsuitable for practical implementation as they are expensive, have long analysis times, and require highly trained personnel. An alternative is the electronic nose (e-nose), which can be used as a non-invasive, rapid, portable piece of equipment that may also provide results at a lower cost per test. An e-nose is an instrument that is designed to sense odours, rather than individually, and typically differentiates them using an array of diverse chemical sensors. There is a wide range of different sensing technologies that can be used inside an e-nose, including surface acoustic wave (SAW) [15,16] quartz crystal microbalance (QCM) [17,18], metal oxide semiconductors (MOS) [19,20,21], conducting polymers (CP) [22], and carbon nanofiber (CNF) [23]. However, MOS-based electronic noses are by far the most common. E-nose technology has previously been used in diverse areas, such as environmental [24,25], food [26,27], pharmaceutical [28,29], biomedical applications [20,30,31,32], and many other fields of applied science.

Many diseases have their own chemical fingerprint, which can be detected by an electronic nose. If we know the chemical fingerprint of a disease, it could be potentially used as a means to identify undiagnosed patients [33,34,35]. Several studies have demonstrated that the e-nose has the ability to detect, differentiate, and identify different cancers. A study was conducted by Di Natale et al. to differentiate between 42 lung cancer patients and 18 healthy controls using breath samples using e-nose based on quartz microbalance (QMB). They were able to identify 100% lung cancer patients and 94% healthy controls [36]. Another study conducted by Westenbrink et al. using a custom e-nose consisting of 13 sensors. They were able to successfully distinguish CRC from Irritable Bowel Syndrome (IBS) using urine samples with a sensitivity of 78% and specificity of 79% [37]. Several other studies have been conducted evaluate the potential of e-nose to differentiate and detect lung cancer [15,38,39,40,41], breast cancer [42], CRC [43,44] and prostate cancer [45,46].

The present study aimed to evaluate the use of PEN3 (Portable Electronic Nose) as a potential e-nose for detection and discrimination between cancer groups and healthy controls. Simultaneously, Gas Chromatography–Time of Flight–Mass Spectrometry (GC-TOF-MS) was used to differentiate cancer group (CRC) from healthy controls and different cancer stages from healthy controls and to determine urinary odour volatile chemical-print for CRC by using the volatile chemical print obtained from urine samples. Urine is commonly used for detection as it is non-invasive and easily obtained from patients. To our knowledge, this is the first study undertaken using the PEN3 applied to the testing of CRC urine samples.

## 2. Materials and Methods

### 2.1. Urine Samples

This study includes the analysis of 96 urine samples acquired at University Hospital Coventry and Warwickshire NHS Trust, after patients provided written informed consent. Out of the 96 samples, there were 58 CRC urine samples and 38 non-cancerous samples. A total of 58 CRC samples were further distributed into 24 early-stage CRC sample and 34 late-stage CRC samples based on TNM (tumour/node/metastasis) staging. We assigned T1 and T2 stage as early-stage and T3 and T4 as late-stage samples. The samples were contained in standard universal sterile specimen containers and frozen within 2 h at −80 °C. This was to allow batch testing of all the samples once they had been collected. The study was approved by Coventry and Warwickshire and North-East Yorkshire NHS Ethics Committees (Ref 18717 and Ref 260179). The samples were later analysed at the University of Warwick. The samples were shipped to the University on dry ice and then stored at −20 °C until analysed, which was within a few days of arrival. For analysis, the samples were defrosted and transferred into 20 mL glass vials with crimp caps. A total of 5 mL of each urine sample was used for the analysis using PEN3 and GC-TOF-MS. The demographic information of the subjects recruited into this study are provided in Table 1. 

### 2.2. PEN3 Electronic Nose (Airsense Analytics GmbH, Schwerin, Germany)

PEN3 (Airsense Analytics GmbH, Schwerin, Germany) is a portable (92 × 190 × 255 mm) olfactory system used for the identification of chemicals and gases. It is a combination of a gas sampling unit and a sensor array. In our case the PEN3 is fitted with an autosampler (HT2000H Dynamic Headspace Auto-sampler, Brescia (BS), Italy), which interfaces directly with the PEN 3 software (WinMuster PEN v 1.6.2.18).

The sensor arrays consist of 10 different thick film metal oxide sensors, operating between 250 and 550 °C. Information available about these sensors are included in Table 2. 

The PEN3 contains two pumps, one is used for pulling the sample gas through the sensor array and the other transfers filtered reference air or zero air into the sensor array. The zero air is also used to clean the system. Zero air is used as a baseline or reference gas, and the sensor response from the sample gas are measured in comparison to the reference gas. Vials containing urine samples were placed in a sample tray of the auto-sampler. These samples were transferred one by one from the sample tray to an internal oven and were heated to increase concentration above the detection limit of the e-nose. Different over and incubation periods were tested, and it was found that with the oven set to 80 °C and the incubation period set to 8 min gave the most consistent results. The oven was equipped with an orbital shaker. After heating up the sample, the headspace was sampled by a syringe at a pressure of 5 bar (max.) and a volume of 2.5 mL. The sample was then analysed for 5 min.

### 2.3. Markes GC-TOF-MS

Markes GC-TOF-MS is a combination of TRACE 1300 GC (Thermo Fisher Scientific, Loughborough, UK) and Bench TOF-HD TOF-MS (Markes Intl., Llantrisant, UK). It consists of a high-throughput autosampler and thermal desorption unit, ULTRA-xr and UNITY-xr, respectively (Markes Intl.). The operating principle of GC-TOF-MS is it analyses the Time of Flight of ions. The GC component separates chemicals according to their interaction with the stationary phase of the column (liquid) and mobile phase (gas) inside the column. TOF-MS separates fragment ions inside the TOF ‘flight box’ and detects them according to the mass-to-charge ratio of the ions after passing through the drift tube.

The sample was transferred on to the TD tube (C2-AXXX-5149, Markes Intl., Llantrisant, UK), by heating 5 mL of urine in a 20 mL glass vial with a crimp cap. The TD is inserted through the septum and into the headspace of the vial. The urine sample is then heated 40 °C for 10 min. After 10 min, a pump (Markes Intl.) is attached to the other end of the TD tube and pulls the urine headspace gas onto the TD tube at a rate of 20 mL/min for a further 10 min. The samples were then analysed by placing the tubes in the autosampler. The analysis was initiated with ULTRA-xr with a stand-by split set to 150 °C and GC run time 25 min with a programmed temperature ramp from 40 °C to 280 °C at 20 °C/min. For each sample, the pre-purge time was 1 min, followed by desorption for 10 min at 250 °C and trap purge for 1 min. These traps were then cooled at −30 °C followed by purging them for 3 min at a temperature of 300 °C [47]. The temperature for both transfer line and ion source was 250 °C. The data obtained was analysed using the national institute of standards and technology (NIST) list (2011).

### 2.4. Statistical Analysis

The data obtained from PEN3 was analysed using MultiSens Analyzer (v2.0.0.22, JLM Innovation GmbH, Germany). MultiSens Analyzer is used to evaluate measurement data from multi sensor instruments, such as electronic noses. MultiSens Analyzer classified the data into different groups and then performed feature extraction. The feature used was the maximum deviation of the signal from the baseline to the response. The resultant data matrix was then analyzed using a 10-fold cross-validation, undertaken using a bespoke R program (version 3.6.2). In a 10-fold cross-validation the dataset is divided into 10 groups. Keeping one of the groups as a test set, the remaining 9 are used as a training set, to which classification models are created and then applied to the test set. For our study, this was Random Forest (using the “Ranger” function in the ‘kernlab’ R package’) and Neural Network (using the “nnet” package). Random forest consists of many decision trees where each tree produces class prediction and the class with the most prediction is assigned as model’s prediction [48]. Neural network classifier consists of layers where first layer is the input, last layer is output, and middle layers are hidden layers. Each layer consists of nodes which converts input into the output [49]. This process was repeated 10 times until all the data had been a test set. These classifiers were chosen as they showed good performance in previous studies [50]. From the resultant probabilities, final statistical results including a Receiver Operator Characteristic (ROC) curve, sensitivity, selectivity, specificity, positive predictive value (PPV), and negative predictive value (NPV) were calculated.

**Figure 1:**
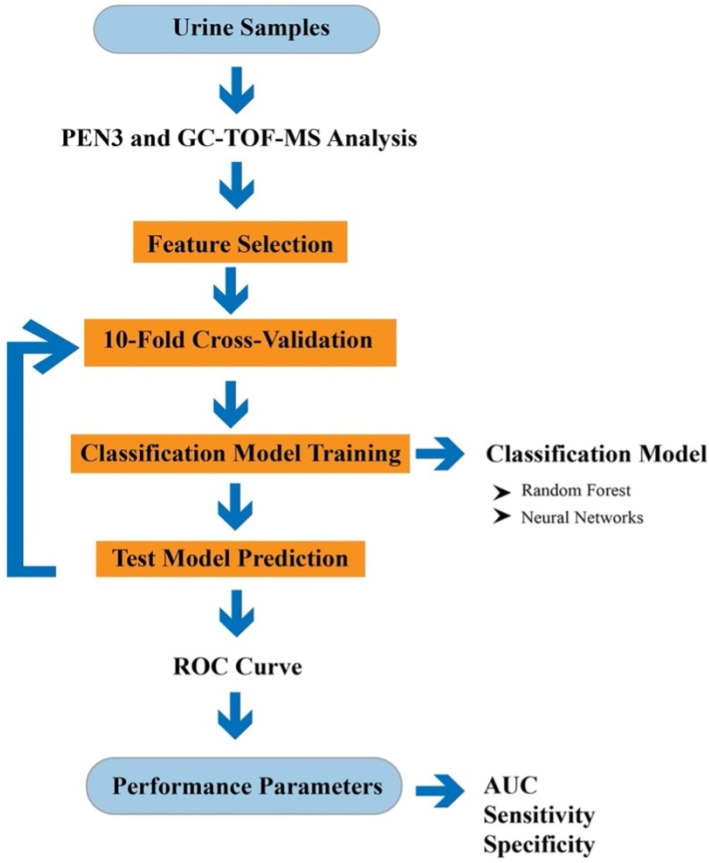
Flowchart of the data analysis used in this study.

For GC-TOF-MS data analysis, the data was processed using the TOF-DS software. A background correction was applied, and the chromatogram was integrated using TOF-DS. The peaks from the chromatogram were identified using the NIST (National Institute of Standards and Technology) list. The TOF-DS software is used to identify chemicals and their abundance in the sample. The chemical identification for GC-TOF-MS data was done based on a *p*-value less than 0.05. These chemicals were then compared with published papers and PubChem. For R analysis, the data obtained from GC-TOF-MS was converted into text files. These files were then used to generate statistical probabilities using R program (version 3.6.2) in a similar process as for the PEN3 and chemical components of discriminative power were identified. Figure 1 illustrates the step taken for analysing the data.

## 3. Results

### 3.1. Evaluation by Electronic Nose Detection Method

**Figure 2:**
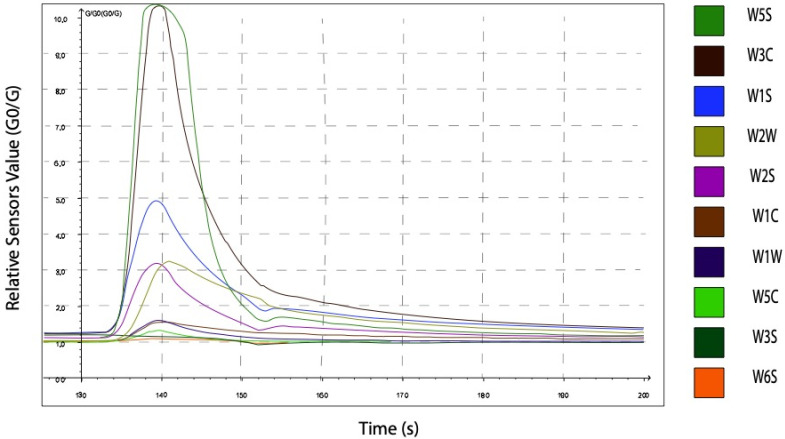
Typical output of the PEN3 to a late CRC urine samples, where each curve representing output from a different sensor.

The PEN3 contains 10 gas sensors, the raw output of each of the sensors to a late CRC urine sample is shown in Figure 2. Each coloured line in the output represents the response curve of one of the sensors.

**Figure 3:**
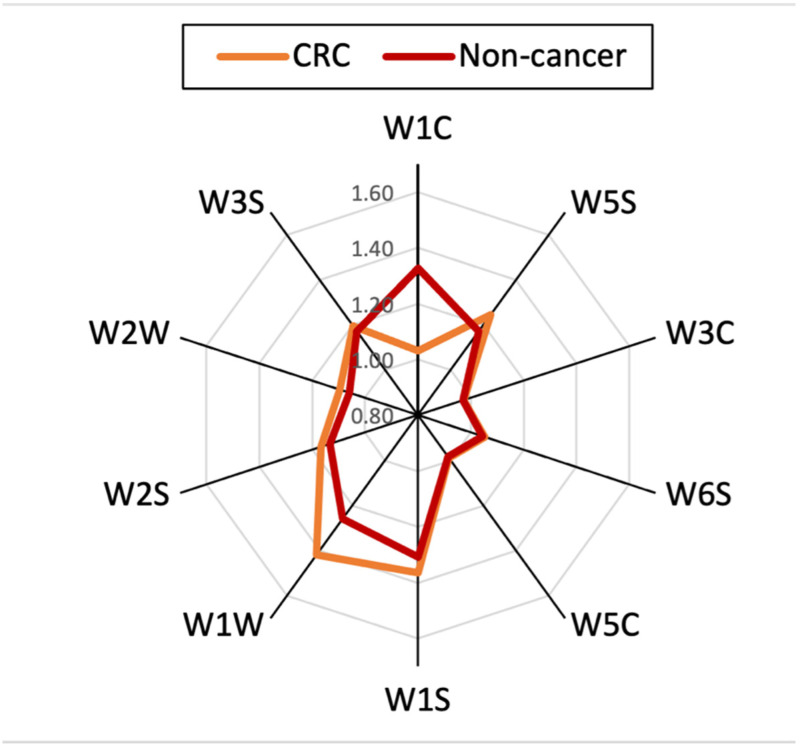
Radar plot of the output features of the PEN3 to a CRC and control sample.

Radar plot shown in Figure 3 represents the average response of PEN3 sensors to the two groups. Radar plot compared sensors response for CRC (red line) and non-cancerous sample (yellow line).

**Figure 4:**
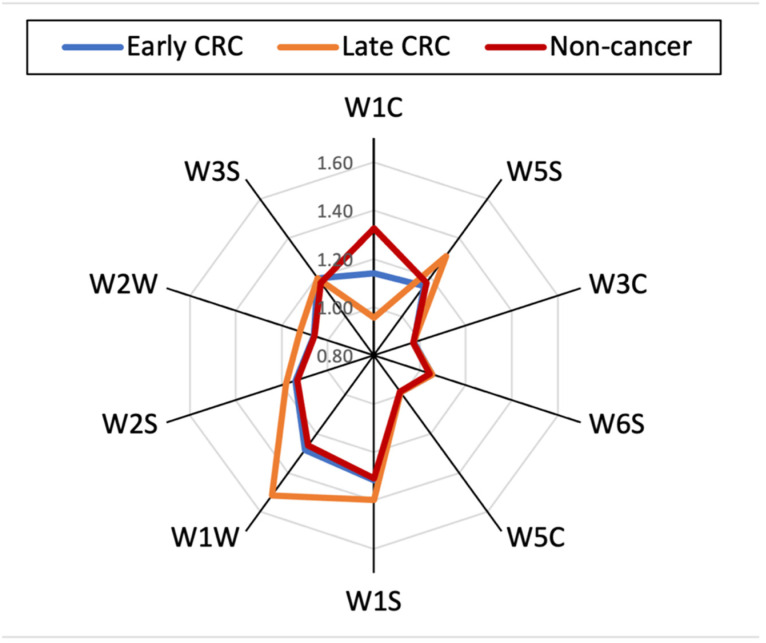
Radar plot of the output features of the PEN3 to an early-stage CRC, late-stage CRC, and control sample.

Radar plot in Figure 4 shows the average response of the sensors corresponding to the early-stage CRC (green line), late-stage CRC (orange line), and non-cancer group (red line).

The results obtained from statistical analysis of PEN3 data validated the separation of the CRC and non-cancerous group based upon the chemicals present in the samples. The results are shown in Table 3.

**Figure 5:**
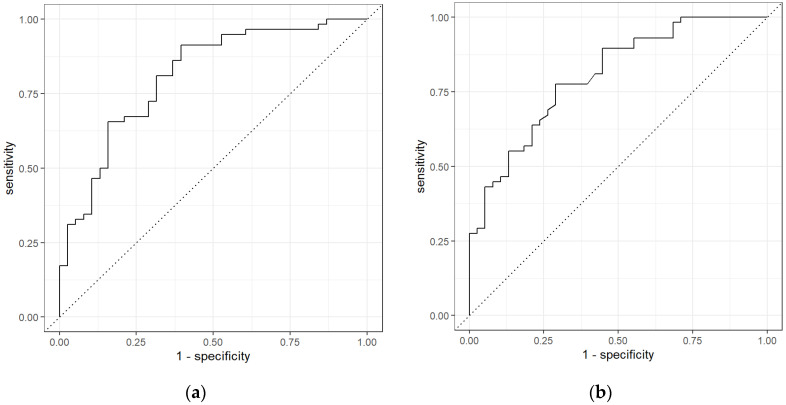
ROC for CRC vs. Non-Cancerous using (**a**) Neural Network and (**b**) Random Forest classifiers for PEN3.

The receiver operating characteristics curves (ROC) of the two models used to obtain highest AUC are shown in Figure 5. The results from R analysis on PEN3 output differentiated CRC from Non-cancerous group with a high sensitivity of 0.91 (0.85–0.97), specificity of 0.55 (0.41–0.69), and AUC of 0.81 (0.73–0.88) using a Neural Network.

### 3.2. Evaluation by GC-TOF-MS Detection Method

**Figure 6:**
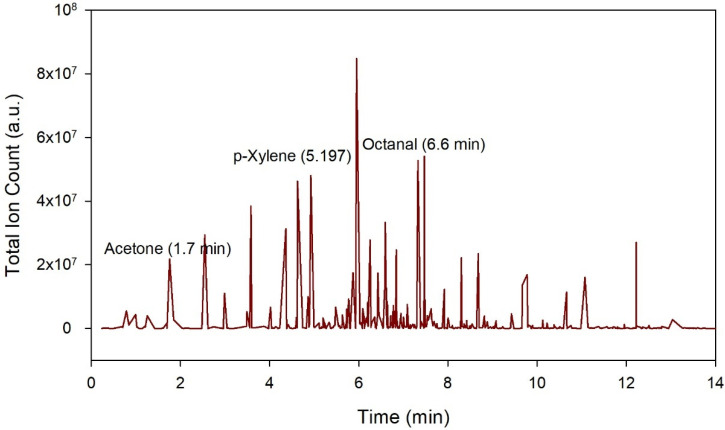
Output from the GC-TOF-MS to a late CRC urine sample with each peak representing the abundance of the chemicals at retention time.

Figure 6 provides an example output from the GC-TOF-MS. Here, the x-axis refers to the retention time, and the y-axis, the total ion count.

GC-TOF-MS shows a very high separation among the two groups, as shown in Table 4. The results illustrate that GC-TOF-MS was able to separate cancer and non-cancerous groups with a very high sensitivity and specificity.

**Figure 7:**
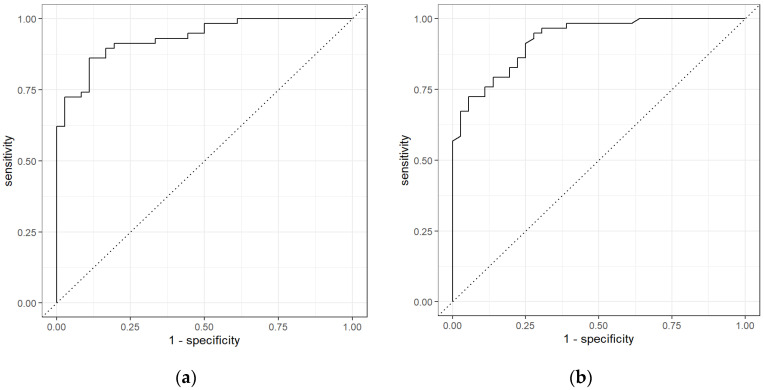
ROC for CRC vs. non-Cancerous using (**a**) Neural Network and (**b**) Random Forest classifiers for GC-TOF-MS.

For CRC and non-cancerous group with Neural network classifier, the sensitivity was 0.86 (0.78–0.93), specificity was 0.86 (0.77–0.95), and AUC was 0.93 (0.89–0.97). Figure 7 represents the ROC curves for GC-TOF-MS.

In addition, data obtained from GC-TOF-MS was used to identify the unknown VOCs in the urine sample headspace. The TOF-DS software identified the chemicals based on NIST list using a criterion of *p*-value < 0.05. We were able to identify 23 VOCs for CRC and non-cancer comparison shown in Table 5. We were able to cross-verify a total of 11 CRC VOCs from different studies and PubChem as shown in Table 5.

Comparisons were also performed on the CRC samples according to the stage of cancer for the quantitative determination of VOCs among the stages. Table 6 illustrates the statistical result obtained for different stages of CRC and non-cancer samples using R analysis using the data obtained from PEN3. Both Random Forest and Neural Network classifiers were used. These gave similar results, with the best result provided in Table 6.

**Figure 8:**
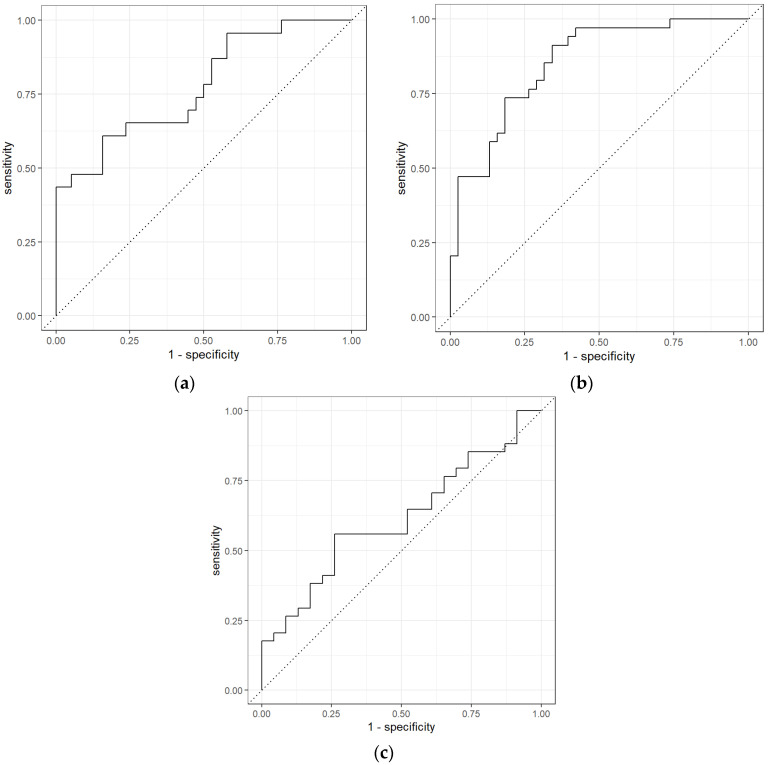
ROC for different stages of CRC vs. non-Cancer: (**a**) early-stage CRC vs. non-cancer using Random Forest classifiers, (**b**) late-stage CRC vs. non-cancer using Neural Network, and (**c**) early-stage CRC vs. late-stage CRC using Neural Network using the PEN3 analytical device.

These results represent that PEN3 was able to distinguish early-stage CRC from non-cancer samples and late-stage CRC from non-cancer samples. Out of 24 early-stage CRC samples, PEN3 was able to identify 14 samples correctly, hence, obtaining a sensitivity of 0.70. While for non-cancer samples, PEN3 recognized 32 out of 38 samples, obtaining a specificity of 0.53. AUC obtained for late-stage CRC and non-cancer samples comparison was 0.85 with a sensitivity and specificity of 0.65 and 0.82, showing that PEN3 identified 22 CRC samples out of 34 and 31 out of 38 non-cancer samples. However, the results for early-stage CRC and late-stage CRC were moderate with 0.61 AUC, 0.68 sensitivity, and 0.39 specificity. The ROC curves for the statistical analysis of different stages of CRC using the data from PEN3 is shown in Figure 8.

The results obtained for the comparison of early-stage CRC, late-stage CRC, and non-cancer samples for GC-TOF-MS data are shown in Table 7.

**Figure 9:**
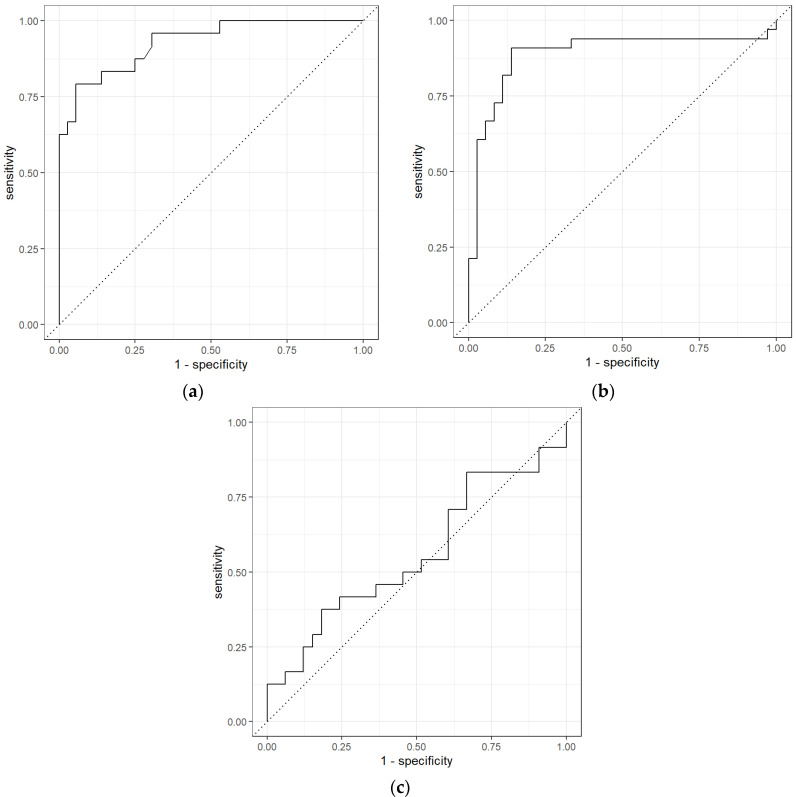
ROC for different stages of CRC vs. non-Cancer: (**a**) early-stage CRC vs. non-cancer using Random Forest classifiers, (**b**) late-stage CRC vs. non-cancer using Neural Network, and (**c**) early-stage CRC vs. late-stage CRC using Neural Network using the GC-TOF-MS analytical device.

For the early-stage CRC vs. non-cancer comparison using GC-TOF-MS, results show that out of 24 early-stage CRC samples, 16 were correctly diagnosed as CRC samples giving sensitivity of 0.67 and out of 38 non-cancer samples, 34 samples were correctly recognized as non-cancer samples giving specificity of 0.94. As for late-stage CRC vs. non-cancer samples, 26 samples out of 34 were correctly diagnosed as CRC samples giving 0.79 sensitivity and 32 samples were correctly recognized as non-caner samples out of 38 giving specificity of 0.89. However, the results obtained for the comparison between early-stage vs. late-stage were moderate. The sensitivity obtained was 0.46 signifying that 11 early-stage samples were correctly diagnosed, and specificity obtained was 0.61 indicating that 20 late-stage CRC samples were correctly diagnosed. Figure 9 shows the ROC curves for these comparisons.

**Figure 10:**
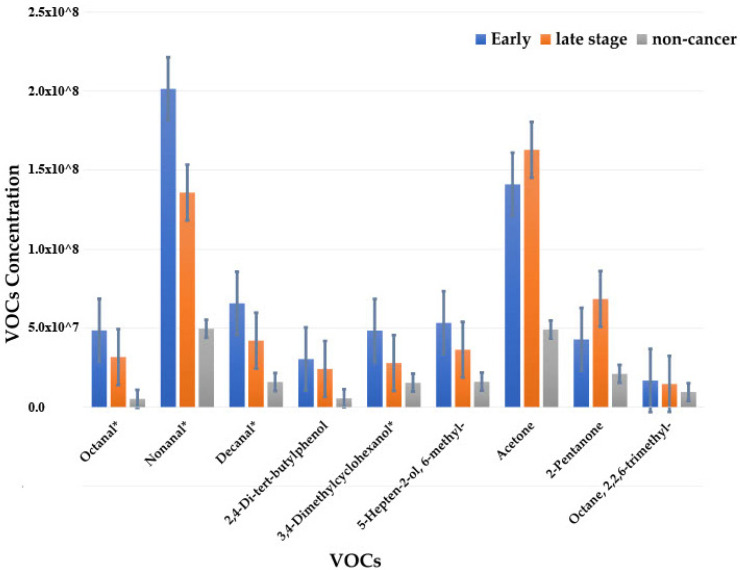
Bar graph for the VOCs present in the study with their respective average concentration among early-stage CRC, late-stage CRC, and non-cancer samples. The * mark represents statistically significant VOCs.

A quantitative comparison of the chemical’s concentration was performed for different stages of CRC and non-cancer samples. Figure 10 illustrates the result obtained. The result shows that the chemicals follow a pattern for early-stage CRC, late-stage CRC, and non-cancer samples.

## 4. Discussion

A number of studies have previously demonstrated the use of urinary headspace VOCs for the detection of different cancers, as well as other diseases [51,52]. Detection of cancer using VOCs is of great interest as it is non-invasive and potentially inexpensive. In this study, we have determined that cancer group can be differentiated from a non-cancer group based on their chemical fingerprints. We used two approaches for distinguishing CRC and the non-cancer group, specifically PEN3 e-nose and GC-TOF-MS. Both methods demonstrated a high accuracy of separation between the groups. We also identified the chemical compounds in the urinary VOC profile for CRC using GC-MS-TOF data.

PEN3 showed promising results with high sensitivity and specificity. The PEN3 was able to differentiate CRC and non-cancer group using Neural Network classifier with AUC of 0.81 and very high sensitivity of 0.91 and specificity of 0.55. The separation between CRC and non-cancer group using Random Forest classifier was reported 0.80 AUC, 0.82 sensitivity and 0.55 specificity.

The AUC obtained by GC-TOF-MS for CRC and non-cancer group was 0.93 with both the classifiers. The sensitivity obtained between the groups was 0.86 for Neural Network classifier and 0.89 for Random Forest. These are very high values showing that GC-TOF-MS was able to recognise and separate CRC and non-cancer urine samples. 

Table 5 shows all the chemicals identified by TOF-DS software of which, octanal, nonanal, decanal, heptanal, hexanal, and acetone had the highest significance with a *p*-value of <0.001. Other possible significant biomarkers were 2-pentanone, 2-heptanone, ethylbenzene, p-xylene, naphthalene.

Octanal is a human metabolite present in cell membrane and generally reported in saliva or faeces [53]. It was reported by Batty et al. on analysis of faecal samples of CRC with PLS-DA following feature selection with Wilcoxon T test [54]. Nonanal is a toxic compound and has been found related with several diseases. It may lead to kidney disease, comas, uraemia, seizures, nausea, confusion, and cardiovascular diseases and has been found in faeces, blood, and saliva from humans [53]. Nonanal has frequently been reported as a breath biomarker for CRC in different studies [55,56,57]. Decanal is another important biomarker observed in our study, which has been reported as a CRC biomarker in several studies as a breath biomarker [55,57] and in cell culture studies [58,59,60]. Heptanal has been identified as a faecal and urinary biomarker for CRC in two different studies [54,61]. Hexanal [55,58] and acetone [44,58,62] were also reported as significant biomarker for CRC.

2-Pentanone is generally present in milk [61,63] and different foods and found in cytoplasm and extracellular places in human body. 2-Pentanone has been reported as CRC biomarkers by Arasaradnam et al. in their study of colorectal cancer using urinary samples [62]. 2-Heptanone exists at cell membrane level inside living species including humans, and outside, it can be found in milk, corns, and peppermints. It causes hepatic encephalopathy [64] and can be found in saliva, faeces, urine, and cerebrospinal fluid [53]. It has been identified as a significant CRC biomarker using cell culture in two different studies [58,60]. Another important biomarker we found was p-Xylene. p-Xylene has several physiological effects such as drowsiness, paralysis, coma, dizziness, anaemia, hypertension, pain, fatigue, headache, depression, and anxiety. It is an air pollutant and environmental contaminant [53]. It is reported as a CRC VOC in three different studies [55,57,65].

Ethylbenzene is a human metabolite present in subcellular level (membrane) in the human body. It causes dizziness, pain, headache, cough, hepatitis, and drowsiness. It is present in tobacco smoke and is water and air pollutant [53]. De Vietro et al. found that ethylbenzene was present in four out of the seven CRC patients breath samples and tissue samples [56]. Furthermore, study conducted by Altomare et al. showed that ethylbenzene was associated with chemical fingerprint of CRC [66]. Naphthalene has also been suggested as a CRC biomarker in PubChem.

For the PEN3, it is not possible to identify specific chemical biomarkers for CRC. It is worth noting that the sensors which are likely to comprise the PEN3 will be sensitive to both inorganic gases as well as VOCs. In fact, one of the sensors that show a significant difference has increased sensitivity to sulphur compounds. In is worth noting that the pre-concentration approach used here for GC-TOF-MS analysis, limits our range of detection to molecules with more than three carbon atoms. Therefore, the chemical components being measured by the PEN3 and by GC-TOF-MS may not be the same. Furthermore, the gas sensors used in the PEN3 are likely to have cross-sensitivity to a range of inorganic gases. Therefore, the response could be associated with VOCs, inorganic gases, or a combination of both. In the future, we hope to analyse further these chemicals to understand their contribution to the instrument’s diagnostic potential.

A comparison was performed between the different stages of CRC in this study. CRC samples were divided into early-stage CRC and late-stage CRC depending on the TNM staging of each sample. Each sample was assigned T1 to T4 stage depending upon the size and/or extent of the tumour. T1 and T2 were grouped as early-stage cancer and T3 and T4 were grouped as late-stage cancer. These comparisons were performed for both PEN3 and GC-TOF-MS data. The results illustrate that PEN3 was able to separate early-stage CRC and late-stage CRC from non-cancer samples with higher statistical output in comparison to the early-stage CRC versus late-stage CRC. A similar pattern was observed for GC-TOF-MS data. Though the results obtained by GC-TOF-MS data showed greater separation with higher sensitivity and specificity. 

However, the results from the comparison of two stages based on concentration of chemicals found in the study demonstrate high capability of GC-TOF-MS to distinguish the two stages of CRC. Figure 9 illustrates that early-stage, late-stage, and non-cancer samples have different level of concentrations of VOCs. The statistically significant chemicals for this separation, i.e., chemicals with a *p*-value of <0.05, were represented by the * mark. This signifies that the detection of the cancer and the stages of the CRC based on VOCs profile may be a possible diagnosis method. The PEN3 was unable to accurately separate early from late-stage cancer. This may be associated with ability of this system to measure the subtle differences between early and late-stage cancer.

The study limitations were relatively small sample size/single centre study and lack of comparison with healthy control group. We compared cancer group with non-cancer group (patients with history of bowel symptoms suggestive of cancer but subsequently excluded). Another limitation was that no chemical identification was undertaken with calibration standards, and we did not attempt to quantify these chemicals. However, many of the chemicals were reported in other studies and therefore, suggests consistency in reporting.

## 5. Conclusions

In our study, we investigated the use of PEN3 and GC-TOF-MS for the analysis of urinary headspace biomarkers for colorectal cancer. We found that both PEN3 and GC-TOF-MS were successful in separating the groups with high AUC. For the PEN3, the highest AUC was seen for CRC and non-cancer group, AUC was 0.81 (0.73–0.88) and GC-TOF-MS demonstrated relatively higher sensitivity and specificity with AUC for CRC and non-cancer group 0.93 (0.89–0.97). The TOF-DS software was then used to investigate the VOCs linked with both the cancers using GC-TOF-MS data. We found a total of 23 VOCs, out of which 11 were cross verified using published papers and PubChem. This VOC profile may support the use of VOCs for the screening of cancer and confirm clinical diagnostic assessments. This will help in avoiding inefficient analytical methods currently used for screening and give better, cheaper, and non-invasive approach for cancer diagnosis and detection. Further, we verified that e-nose can be used for the detection and diagnosis of cancer as it demonstrates high sensitivity and specificity.

## Figures and Tables

**Table 1:** Clinical characteristics of the recruited study participants at time of obtaining the urine samples.

Group	CRC	Non-Cancerous
Number of samples	58 (24 early stage and 34 late stage)	38
Mean Age (years)	74.7 (92–46)	63.2 (90–32)
Sex: Male/Female	40:18	25:13
Avg. BMI	27.7 (34.1–17)	30.5 (37.3–22.4)
Current Smoker (Number and % of patients)	3 (5.2%)	3 (8.3%)

**Table 2:** Description of the sensors used in PEN3 e-nose provided by Airsense Analytical.

Sensor No.	Sensors	Substances for Sensing
S1	W1C	Sensitive to aromatic compounds
S2	W5S	Broad range
S3	W3C	Sensitive to aromatic compounds
S4	W6S	Sensitive to hydrogen
S5	W5C	Sensitive to aromatic and aliphatic compounds
S6	W1S	Sensitive to methane in the environment, with broad range
S7	W1W	Sensitive to Sulphur and organic compounds
S8	W2S	Sensitive to alcohol and broad range
S9	W2W	Sensitive to Sulphur compounds
S10	W3S	Sensitive to methane and aliphatic compounds

**Table 3:** Statistical Results for PEN3 with 95% confidence intervals in brackets.

Classifiers	Comparisons	AUC	Sensitivity	Specificity	PPV	NPV
Neural Network	CRC vs. Non-Cancerous	0.81 (0.73–0.88)	0.91 (0.85–0.97)	0.55 (0.41–0.69)	0.76 (0.61–0.84)	0.81 (0.67–0.93)
Random Forest	CRC vs. Non-Cancerous	0.80 (0.72–0.87)	0.82 (0.74–0.90)	0.55 (0.41–0.68)	0.74 (0.65–0.83)	0.68 (0.53–0.82)

**Table 4:** Statistical Results for GC-TOF-MS with 95% confidence intervals in brackets.

Classifiers	Comparisons	AUC	Sensitivity	Specificity	PPV	NPV
Neural Network	CRC vs. Non-Cancerous	0.93 (0.89–0.97)	0.86 (0.79–0.93)	0.81 (0.77–0.95)	0.91 (0.84–0.97)	0.79 (0.69–0.89)
Random Forest	CRC vs. Non-Cancerous	0.93 (0.88–0.96)	0.89 (0.83–0.96)	0.75 (0.62–0.86)	0.85 (0.77–0.92)	0.82 (0.71–0.93)

**Table 5:** VOCs obtained from GC-TOF-MS data analysis using the TOF-DS software.

S. No.	Chemicals	Retention time (s)	*p*-Value
1	Octanal ^a^	6.5851	<0.001
2	Nonanal ^a^	7.4723	<0.001
3	Decanal ^a^	7.5505	<0.001
4	2,4-Di-tert-butylphenol	10.6607	<0.001
5	Heptanal ^a^	5.6302	<0.001
6	Heptadecane	10.3784	<0.001
7	Undecanal	9.08	<0.001
8	3,4-Dimethylcyclohexanol	9.6693	<0.001
9	5-Hepten-2-ol, 6-methyl-	9.7753	<0.001
10	Hexanal ^a^	4.6015	<0.001
11	Acetone ^a^	1.6974	<0.001
12	2-Pentanone ^a^	3.4864	0.001
13	Biphenyl	9.7844	0.003
14	2-Heptanone ^a^	5.5489	0.00429
15	Cyclopentanone, 2-methyl-	5.772	0.00453
16	Ethylbenzene ^a^	5.107	0.00499
17	Methane, isocyanato-	1.4175	0.00666
18	Acetophenone	7.3934	0.00888
19	1-Undecanol	9.6614	0.01307
20	p-Xylene ^a^	5.197	0.01478
21	Benzene, 1-methyl-3-(1-methylethyl)-	6.6649	0.01602
22	Naphthalene ^a^	8.3504	0.02613
23	Octane, 2,2,6-trimethyl-	5.8677	0.04052

^a^ represents the chemicals for the identification of CRC cross-verified using PubChem and published papers.

**Table 6:** Statistical Results for CRC stage comparisons with 95% confidence intervals in brackets using PEN3.

Classifiers	Comparisons	AUC	Sensitivity	Specificity	PPV	NPV
Neural Network	Early vs. Non-Cancer	0.67 (0.54–0.79)	0.48 (0.30–0.65)	0.84 (0.74–0.93)	0.65 (0.44–0.83)	0.73 (0.61–0.83)
Random Forest	Early vs. Non-Cancer	0.78 (0.66–0.87)	0.61 (0.43–0.76)	0.84 (0.74–0.94)	0.70 (0.52–0.86)	0.78 (0.67–0.88)
Neural Network	Late vs. Non-Cancerous	0.85 (0.78–0.92)	0.65 0.51–0.78)	0.82 (0.71–0.92)	0.76 (0.63–0.89)	0.72 (0.61–0.83)
Random Forest	Late vs. Non-Cancerous	0.76 (0.66–0.84)	0.74 (0.61–0.85)	0.66 (0.53–0.79)	0.66 (0.53–0.79)	0.74 (0.61–0.85)
Neural Network	Early vs. Late CRC	0.61 (0.49–0.73)	0.68 (0.54–0.81)	0.39 (0.22–0.56)	0.62 (0.49–0.75)	0.45 (0.26–0.64)
Random Forest	Early vs. Late CRC	0.59 (0.45–0.71)	0.71 (0.57–0.83)	0.39 (0.22–0.56)	0.63 (0.50–0.76)	0.48 (0.28–0.67)

**Table 7:** Statistical Results for CRC stage comparisons with 95% confidence intervals in brackets using GC-TOF-MS.

Classifiers	Comparisons	AUC	Sensitivity	Specificity	PPV	NPV
Neural Network	Early vs. Non-Cancer	0.9 (0.83–0.96)	0.75 (0.59–0.89)	0.86 (0.76–0.95)	0.78 (0.64–0.92)	0.84 (0.73–0.93)
Random Forest	Early vs. Non-Cancer	0.93 (0.87–0.96)	0.67 (0.5–0.82)	0.94 (0.88–1)	0.89 (0.75–1)	0.81 (0.71–0.90)
Neural Network	Late vs. Non-Cancerous	0.89 (0.81–0.96)	0.79 (0.67–0.90)	0.89 (0.79–0.97)	0.87 (0.76–0.97)	0.82 (0.72–0.92)
Random Forest	Late vs. Non-Cancerous	0.86 (0.78–0.93)	0.73 (0.59–0.83)	0.81 (0.68–0.91)	0.77 (0.64–0.89)	0.76 (0.65–0.8)
Neural Network	Early vs. Late CRC	0.56 (0.43–0.69)	0.46 (0.29–0.63)	0.61 (0.46–0.74)	0.46 (0.29–0.62)	0.61 0.43–0.74)
Random Forest	Early vs. Late CRC	0.56 (0.43–0.69)	0.38 (0.22–0.55)	0.69 (0.56–0.83)	0.47 (0.29–0.67)	0.61 (0.43–0.74)

## Data Availability

All data are available from this manuscript.
